# 原发纵隔大B细胞淋巴瘤的治疗进展

**DOI:** 10.3760/cma.j.cn121090-20230731-00041

**Published:** 2024-01

**Authors:** 吕雯 陈, 建勇 李, 磊 范

**Affiliations:** 南京医科大学第一附属医院，江苏省人民医院血液科，南京 210029 Department of Hematology, the First Affiliated Hospital with Nanjing Medical University, Jiangsu Province Hospital, Nanjing 210029, China

## Abstract

原发纵隔大B细胞淋巴瘤（PMBCL）是一种起源于胸腺的侵袭性B细胞淋巴瘤。具有不同于非特指型弥漫大B细胞淋巴瘤的临床和生物学特征，PMBCL好发于年轻女性，通常表现为前纵隔巨大肿块，大多数患者就诊时处于Ⅰ～Ⅱ期，目前尚无标准的PMBCL预后评分系统。PMBCL治疗多采用免疫化疗，但最佳一线治疗方案尚无定论，并且放疗的地位存在争议，PET-CT指导治疗的价值有待进一步证实。复发/难治PMBCL预后较差，PD-1抑制剂、维布妥昔单抗和CAR-T细胞等新型疗法有助于改善此类患者生存。

## Abstract (en)

Primary mediastinal large B-cell lymphoma (PMBCL) is an aggressive B-cell lymphoma originating from the thymus, which has different clinical and biological characteristics from diffuse large B-cell lymphoma, NOS. PMBCL tends to occur in young women, usually presenting as a large anterior mediastinal mass. Most patients are in stage Ⅰ-Ⅱ at the time of presentation. There is no standard prognostic scoring system for PMBCL. Immunochemotherapy is commonly used in the treatment of PMBCL, but the optimal first-line treatment has not been determined, and the status of radiotherapy is controversial. The value of PET-CT guided therapy needs to be further verified. Relapsed/refractory PMBCL has a poor prognosis, while novel therapies such as PD-1 inhibitors, brentuximab vedotin, and CAR-T can help improve survival in these patients.

原发纵隔大B细胞淋巴瘤（PMBCL）是弥漫大B细胞淋巴瘤（DLBCL）的一种特殊亚型，在2016年世界卫生组织（WHO）对恶性淋巴瘤的分类中，确定PMBCL是一个独立的临床和病理亚型[1]。PMBCL是一种起源于胸腺髓质B细胞的侵袭性淋巴瘤，占非霍奇金淋巴瘤（NHL）的2％～4％，占DLBCL的7％[2]。PMBCL临床和生物学特征与非特指型（NOS）DLBCL不同，而与结节硬化型经典型霍奇金淋巴瘤更加相似。关于PMBCL的治疗，目前仍有许多问题亟待解决，包括预后评分系统、最佳一线治疗方案、放疗的地位和价值、PET-CT指导治疗的作用，以及复发/难治（R/R）PMBCL患者的治疗。本文就近年来PMBCL的研究进展及以上问题进行综述。

一、临床特征

PMBCL好发于30～40岁的年轻女性，男女比例为1∶3[3]。PMBCL通常表现为迅速进展的前纵隔巨大肿块（>10 cm），常累及周围脏器，如肺、胸膜和心包等，可导致胸水和心包积液的产生。局部肿瘤压迫可使患者出现呼吸困难、咳嗽、吞咽困难、声音嘶哑、气道阻塞，可出现上腔静脉综合征，表现为胸部和颈部静脉扩张、面部水肿、结膜充血，偶尔出现手臂肿胀[4]。大约80％患者在诊断时处于Ⅰ期或Ⅱ期[2]，约三分之一的患者可出现B症状（发热、体重减轻、盗汗）[5]。70％～80％的初诊PMBCL患者LDH升高[6]–[8]。初诊时病变大多局限于胸腔内，偶有锁骨上淋巴结受累，但很少累及胸腔以外和结外器官。如果疾病复发进展，病变可扩散至全身其他部位，如肾脏、肾上腺、肝脏、卵巢和中枢神经系统，但骨髓受累少见。

二、病理及分子生物学特征

组织学上，PMBCL典型表现为弥漫性生长的、中等到大的肿瘤细胞，细胞质丰富，细胞核为圆形或卵圆形，有时可见多形性细胞核，与HL的里-施（RS）细胞相似，伴有不同程度的纤维化，周围包绕的胶原纤维将肿瘤细胞分隔[9]。

在免疫表型方面，PMBCL通常表达B细胞相关抗原，如CD19、CD20、CD22、CD79a、PAX5和CD45，但常缺乏膜表面和胞质免疫球蛋白表达。大约80％病例可弱表达CD30。Bcl-2、CD23常呈阳性，部分表达Bcl-6和CD15，CD10常为阴性。T淋巴细胞成熟相关蛋白（MAL）在大约70％的PMBCL病例中呈阳性，而在DLBCL和经典型霍奇金淋巴瘤中偶尔表达[10]。

JAK-STAT和NF-κB信号通路中重现性遗传学异常导致其过度激活是PMBCL标志性分子遗传改变。50％～70％的PMBCL患者存在9p24染色体扩增，使得JAK2重复扩增，并正向调节程序性死亡配体-1（PD-L1）和程序性死亡配体-2（PD-L2）的表达，导致JAK-STAT通路失调，从而使肿瘤细胞得以免疫逃逸。染色体2p16扩增导致编码核转录因子NF-κB家族成员之一的原癌基因c-REL激活，从而促进肿瘤细胞生长，并导致其凋亡受抑[5],[10]。因此，JAK-STAT通路和NF-κB可能是PMBCL潜在的治疗靶点。Steidl等[11]在约40％ PMBCL患者样本中发现CIITA易位，这种易位将CIITA的N末端与其他多种基因融合，造成CIITA的一个拷贝失活，MHC Ⅱ类蛋白表达沉默，从而使肿瘤细胞与T细胞相互作用的能力得到限制，表明CIITA重排是PMBCL免疫逃逸的另一重要机制。2021年第63届美国血液学协会（ASH）年会上报道在67％融合阳性的PMBCL病例中检测到新的伴侣基因IGHA1、IGHG1和CDC6。此外，该研究还发现了NOTCH4、DICER1、MCL1等新型候选驱动基因的重现性体细胞变异[12]。Liu等[13]分析了PMBCL患者中在WPD环、P环和Q环上发生突变的3种PTP1B突变的分子及功能水平，发现这些突变导致蛋白质酪氨酸磷酸酶活性丧失，产生异常细胞信号传导，可能参与PMBCL的发生发展过程。Pang等[14]发现STAT6、SOCS1、TNFAIP3基因与R/R PMBCL有关。近期有研究在R/R PMBCL患者中发现了FGFR3、PIK3CA、ALK、EZH2、RNF43和MET基因的错义突变，并提出FGF/FGFR3是R/R PMBCL重要的信号通路[15]。

三、预后因素

由于PMBCL通常为年轻患者，病灶多局限在纵隔，因此淋巴瘤国际预后指数（IPI）和年龄调整后的IPI（aaIPI）对PMBCL患者的预后评价有限[16]，约一半患者在发病时的IPI评分较低。有研究报道Ⅳ期疾病、较高IPI、B症状和Ki-67≥70％是PMBCL患者的不良预后因素[16]–[17]，而MUM1阳性表达和较高外周血淋巴细胞/单核细胞比率分别与较长的总生存（OS）期和无进展生存（PFS）期明显相关[16]。Steiner等[18]通过多重免疫荧光（mIF）分析免疫细胞亚群发现，在治疗前的活检样本中，PD-L1阳性巨噬细胞百分比高、CD30阳性PD-L1阳性细胞和CD30阳性细胞密度高的患者，在治疗开始后的12个月内复发风险较低。

PET-CT对PMBCL疾病分期具有重要价值，近年来多项研究表明PET-CT相关参数对疾病预后具有重要预测价值。Camus等[19]发现基线总肿瘤代谢体积（TMTV）≥360 cm^3^与较差OS相关。Zhou等[16]发现基线、中期及结疗时较高PET最大标准化摄取值（SUVmax）与较差预后有关。Pinnix等[20]发现基线总病变糖酵解（TLG）和Deauville评分与较差的PFS相关。国际结外淋巴瘤研究组（IELSG）开展的IELSG-26研究前瞻性纳入了103例PMBCL患者，分析了代谢异质性（MH）的预后价值，结果显示低MH组和高MH组的5年PFS率分别为94％和73％，差异具有统计学意义[21]。

近年来研究发现分子异常也是PMBCL重要预后指标。有研究报道3例（75％）复发病例在影像学表现为复发的前半个月就检测到循环肿瘤DNA（ctDNA）存在，提示基于ctDNA动态监测可以帮助早期预测复发[14]。2022年第27届欧洲血液学协会（EHA）年会上一项纳入329例PMBCL患者的多组学分析研究显示，存在CD58突变患者生存期明显较短，而发生DUSP2突变患者则显示更长的生存期，且在DUSP2突变这类低风险患者群体中使用CHOP和强化方案治疗预后差异无统计学意义[22]。

四、治疗

1. 一线系统性治疗：由于缺乏前瞻性随机对照临床试验，PMBCL最佳一线治疗方案目前仍然存在争议，加之R/R PMBCL的预后欠佳，因此初始治疗对预后十分重要。含蒽环类药物的化疗方案是PMBCL最常使用的方案。Lisenko等[23]通过回顾性分析发现CHOP方案加利妥昔单抗可以显著改善PMBCL患者预后。Vassilakopoulos等[24]认为结外病变/Ⅳ期、大块疾病和LDH≥2倍正常值上限（ULN）可定义为高风险亚组，而没有以上风险因素则有助于确定非高危患者亚组并使用R-CHOP方案充分治疗，避免不必要的强化治疗。

近年来，以DA-EPOCH-R为代表的强化治疗方案开始受到关注。Dunleavy等[6]进行的一项单臂Ⅱ期前瞻性研究显示，共51例PMBCL患者使用DA-EPOCH-R方案治疗，5年无事件生存（EFS）率为93％，5年OS率为97％。一项多中心回顾性分析发现，R-CHOP和DA-EPOCH-R方案的2年OS率之间差异无统计学意义（89％对91％），但DA-EPOCH-R方案的完全缓解率（CRR）优于R-CHOP方案（84％对70％）[25]。一项来自4个国家225例患者接受DA-EPOCH-R方案治疗的研究显示，5年OS率为92％，5年无进展生存（PFS）率为86％，略优于此前所报道R-CHOP的生存数据，但差异无统计学意义[26]。

一项Ⅰ/Ⅱ期多中心试验使用6个周期维布妥昔单抗（BV）联合利妥昔单抗、环磷酰胺、阿霉素和泼尼松（R-CHP）作为一线方案治疗CD30阳性B细胞淋巴瘤患者。在22例PMBCL患者队列中，2年PFS率为86％，2年OS率为100％。表明BV联合R-CHP是治疗PMBCL可行且有效的一线方案[7]。

2. 巩固放疗的地位：PMBCL中放疗的地位目前仍然存在争议，PMBCL肿瘤大多为纵隔部位的大包块病变，所以临床上大多经验性使用局部放疗巩固。但由于PMBCL通常影响年轻人群，以女性居多，放疗的长期毒性，包括继发恶性肿瘤（如乳腺癌、肺癌）、心血管系统和肺部疾病等限制了放疗在临床上的应用。Avilés等[27]进行的一项随机对照试验表明，经过6个周期R-CHOP方案治疗后，通过增强CT评估，324例PMBCL患者达到完全缓解（CR），以1∶1的比例随机分配到放疗组和对照组，结果显示放疗组与对照组的5年PFS率分别为84％和67％，5年OS率分别为86％和68％，差异均有统计学意义。由于该项试验使用的是增强CT对患者疗效进行评估，评估准确性存疑。另一项使用R-CHOP方案的前瞻性随机试验亚组分析显示，被分配到放疗组的患者3年EFS率较好（94％对78％），但PFS和OS在放疗组和对照组之间差异无统计学意义[28]。一项基于美国人群的回顾性研究显示，放疗可为40～59岁的PMBCL患者群体提供生存获益，而在18～39岁的患者中，放疗与预后的关系并不显著，可以考虑在该年龄段的PMBCL群体中省略放疗[29]。临床上应充分考虑化疗方案强度、疾病缓解程度、疗效评估方式等因素，决定免疫化疗后是否进行巩固放疗。

3. PET-CT指导治疗的价值：一项190例PMBCL患者的研究中，治疗结束（EOT）后136例（71.6％）获得PET阴性缓解（PET-neg），43例（22.6％）PET阳性（PET-pos），11例（5.8％）PET结果不确定（PET-un），三组的5年OS率依次为89.4％、80.0％、70.0％。在PET-neg患者中，54例（39.0％）使用了放疗，83例（61.0％）继续观察，放疗组与观察组5年PFS和OS率无差异（95.5％对85.4％；97.6％对90.2％）[30]。意大利一项DA-EPOCH-R方案治疗研究发现，28例（70％）EOT-PET阴性，12例（30％）阳性。15例（37％）接受了巩固性放疗。EOT-PET扫描阴性者的PFS和OS率为100％。在EOT-PET扫描阳性的患者中，4年估计PFS率为53％，4年估计OS率为73％[31]。以上结果表明EOT-PET是PMBCL疗效评估重要工具，有助于临床做出是否进一步巩固放疗的决定，以尽可能减少放疗的使用。正在进行的一项前瞻性随机临床研究IELSG-37有望进一步确定PET-CT指导放疗的价值。

4. R/R PMBCL的治疗：

（1）造血干细胞移植：欧洲一项回顾性研究显示，44例R/R PMBCL患者进行自体造血干细胞移植（auto-HSCT），3年PFS率和OS率分别为64％和85％[32]。另一项纳入60例R/R PMBCL患者的研究显示，51例（85％）接受auto-HSCT的患者估计3年OS率和EFS率分别为68％和65％[33]。一项纳入33例R/R PMBCL异基因造血干细胞移植的回顾性研究结果显示，移植时50％患者CR，40％患者部分缓解（PR），10％患者疾病进展（PD）。整个队列的2年OS率、PFS率、无移植物抗宿主病/无复发生存率（GRFS）分别为48％、47％、38.5％。移植时PD的患者2年PFS率和OS率最差[34]–[35]。但是目前对于auto-HSCT在PMBCL患者一线巩固治疗中的地位缺乏相关研究。

（2）PD-1/PD-L1抑制剂：大部分PMBCL患者存在9p24染色体扩增，导致PD-L1/L2过度表达，为PD-1/PD-L1抑制剂的使用提供了理论基础。Casadei等[36]一项回顾性研究显示，18例R/R PMBCL患者接受了帕博利珠单抗治疗，6例（30％）获得了CR，中位随访18个月后，6例患者仍处于持续CR状态。Keynote-170研究结果显示，53例R/R PMBCL患者接受帕博利珠单抗治疗，客观缓解率（ORR）为41.5％，CRR为20.8％，PR率为20.8％。中位PFS期为4.3个月，48个月PFS率为33.0％；中位OS期为22.3个月，48个月OS率为45.3％[37]。以上结果表明帕博利珠单抗治疗表现出持续的抗肿瘤活性，PFS和OS显示出长期生存趋势。

（3）维布妥昔单抗：意大利一项单臂、多中心、Ⅱ期临床试验评估BV单药治疗15例R/R PMBCL患者发现，15例患者中有2例达到PR，1例疾病稳定（SD），其余12例PD，ORR为13.3％[38]。一项Ⅱ期CheckMate 436研究评估了30例R/R PMBCL患者使用纳武利尤单抗（NIVO）联合BV的治疗效果，中位随访时间为 33.7 个月，ORR为73％，CRR为37％，中位缓解持续时间（DOR）为31.6个月且未达中位CR时间持续时间。预期中位PFS期为26.0个月，24个月OS率为76％[39]。一项回顾性研究中，9例R/R PMBCL患者接受NIVO联合BV方案治疗，4例（44.4％）获得CR，中位随访10个月后，这4例患者均处于持续CR状态[36]。以上研究表明，BV单药治疗R/R PMBCL疗效不佳，但NIVO+BV方案表现出持久反应和长期生存益处。

（4）CAR-T细胞治疗：ZUMA-1单臂、多中心Ⅰ/Ⅱ期研究显示，108例难治性大B细胞淋巴瘤患者接受了阿基伦赛的CAR-T细胞治疗，其中包括8例R/R PMBCL患者。中位随访27.1个月，整体队列ORR为83％，CRR为58％，中位DOR为11.1个月，中位OS未达到，中位PFS期为5.9个月[40]。另一项美国的TRANSCEND-NHL-001多中心研究中，344例患者接受Lisocabtagene maraleucel的CAR-T细胞治疗，14例R/R PMBCL患者的ORR为79％，其中7例（50％）达到CR[41]。以上研究显示CAR-T细胞治疗在多重治疗失败的R/R PMBCL患者中有一定疗效。而当CAR-T细胞治疗失败后，R/R PMBCL预后极差，需进一步探索相应的挽救策略。2022年第64届ASH会议上报道了一项研究，共纳入49例抗CD19 CAR-T细胞治疗失败后的R/R B-NHL患者，其中包括9例PMBCL患者，接受二次CAR-T细胞治疗组的3个月PFS率（48.0％对34.4％）和6个月OS率（53.1％对34.52％）比联合化疗组更长[42]。另一项关于成年B细胞淋巴瘤患者CAR-T细胞治疗复发后接受PD-1/PD-L1抑制剂治疗的回顾性研究中，共纳入96例患者，其中包括51例（53％）DLBCL患者，8例（8％）PMBCL患者，PMBCL患者的ORR为63％，明显高于DLBCL患者（12％），但OS没有得到改善[43]。检查点抑制剂（CPI）治疗在CAR-T细胞治疗失败后的作用有望进一步在真实世界中进行研究。

五、结语

PMBCL是一种独特临床和病理类型的侵袭性B细胞淋巴瘤，主要发生在年轻女性。DA-EPOCH-R方案在初诊PMBCL患者中疗效较好，基于PET-CT评估诱导治疗达到完全代谢缓解（CMR）的患者可以暂不考虑纵隔放疗。大部分PMBCL患者可以通过一线治疗获得治愈，一旦疾病复发，患者预后较差。对于R/R PMBCL，auto-HSCT显示了较好的生存获益。PMBCL生物学方面的研究进展为靶向治疗药物的应用提供基础，PD-1抑制剂、维布妥昔单抗和CAR-T细胞疗法为R/R PMBCL患者带来希望，有望进一步改善此类患者临床预后。
